# Serum homocysteine and folate concentrations in early pregnancy and subsequent events of adverse pregnancy outcome: the Sichuan Homocysteine study

**DOI:** 10.1186/s12884-020-02860-9

**Published:** 2020-03-18

**Authors:** Chenggui Liu, Dan Luo, Qin Wang, Yan Ma, Longyu Ping, Ting Wu, Jian Tang, Duanliang Peng, PingZhao

**Affiliations:** 1 Department of Clinical Laboratory, Chengdu Women’s and Children’s Central Hospital, School of Medicine, University of Electronic Science and Technology of China, Chengdu, 611731 China; 2 Chengdu, China; 3 Department of Obstetrics, Chengdu Women’s and Children’s Central Hospital, School of Medicine, University of Electronic Science and Technology of China, Chengdu, 611731 China; 4 Department of Pediatrics, Sichuan Academy of Medical Sciences and Sichuan Provincial People’s Hospital, School of Medicine, University of Electronic Science and Technology of China, Chengdu, 611731 China; 5 Department of Clinical Laboratory, Mianyang Hospital of Traditional Chinese Medicine, Mianyang, 621000 China; 6 Department of Nutrition, Chengdu Women’s and Children’s Central Hospital, School of Medicine, University of Electronic Science and Technology of China, Chengdu, 611731 China; 7 Department of Clinical Laboratory, Sichuan Academy of Medical Sciences and Sichuan Provincial People’s Hospital, School of Medicine, University of Electronic Science and Technology of China, Chengdu, 611731 China; 8 Department of Obstetrics and Gynecology, Mianyang Hospital of Traditional Chinese Medicine, Mianyang, 621000 China

**Keywords:** Adverse pregnancy outcome, Early pregnancy, Folate, Homocysteine, Vitamin B12

## Abstract

**Background:**

Hyperhomocysteinemia may be a risk factor for endothelial dysfunction. Folate and vitamin B12 regulate the homocysteine metabolic process. This study aimed to evaluate the associations between subsequent events of adverse pregnancy outcome and early variables of homocysteine, folate, and vitamin B12 in pregnant women.

**Methods:**

This multicenter, retrospective, case–control study involved 563 pregnant women with adverse pregnancy outcome and 600 controls. Adverse pregnancy outcomes included one or more of the following events: preeclampsia, preterm birth, low birth weight, and stillbirth. The associations between subsequent events of adverse pregnancy outcome and early variables of homocysteine, folate, and vitamin B12; metabolic parameters; inflammatory markers; anthropometrics; and lifestyle habits at 11–12 weeks of gestation were analyzed using the logistic regression model.

**Results:**

Compared to the lower quartile homocysteine concentrations, the upper quartile homocysteine concentrations were associated with preeclampsia, preterm birth and low birth weight. On the contrary, the lower quartile folate concentrations were associated with preeclampsia, preterm birth and low birth weight compared with the upper quartile folate concentrations. The incidence of adverse pregnancy outcome increased progressively from the first to fourth homocysteine quartiles but decreased progressively from the first to fourth folate quartiles. After adjusting for confounding factors, multivariate logistic regression analysis showed that besides systolic blood pressure, diastolic blood pressure, body mass index and age, homocysteine (IV vs I quartile, aOR 5.89, 95% CI 4.08–8.51, *P* < 0.001), folate (IV vs I quartile, aOR 0.35, 95% CI 0.25–0.50, *P* < 0.001), folate supplementation (yes vs no, aOR 0.55, 95% CI 0.35–0.86, *P* = 0.010) during early pregnancy were independently associated with subsequent events of adverse pregnancy outcome, and vitamin B12 was rejected. Of these, the homocysteine revealed the highest odds ratio in all risk variables, and folate showed the lowest odds ratio in all protective variables.

**Conclusions:**

Higher homocysteine concentration and lower folate level during early pregnancy were associated with adverse pregnancy outcome. However, no association was found between vitamin B12 and adverse pregnancy outcome. Supplementation with folate in early pregnancy may reduce adverse pregnancy outcome.

## Background

Adverse pregnancy outcome (APO) is defined as an event that reduces the opportunity of having a healthy newborn, including preeclampsia, preterm birth, low birth weight, stillbirth, and so on. APOs may lead to severe complications, such as massive postpartum hemorrhage and neonatal death [1, 2]. Preeclampsia is a serious, pregnancy-specific syndrome that affects multiple organs. It continues to afflict 5–8% of pregnancies worldwide and is one of the leading causes of morbidity and mortality among pregnant women and fetuses [3]. According to the World Health Organization (WHO), low birth weight is defined as a birth weight less than 2500 g regardless of gestational age, which may be due to either preterm birth or intrauterine growth restriction (IUGR), or a combination of both [4]. The burden of APO is substantial in both developed and developing countries. It is not only the primary cause of infant morbidity and mortality but also the critical determinant of child survival, disabilities, stunting, and long-term adverse consequences [5, 6].

Previous studies demonstrated that elevated levels of homocysteine (Hcy) were associated with an increased risk of vascular diseases and may cause direct damage to endothelial cells both in vitro and in vivo [7, 8]. Maternal hyperhomocysteinemia (HHcy) exhibited a wide range of effects, including maternal and placental vascular endothelial dysfunction, DNA dysfunction, and proliferation of smooth muscle cells through oxidative stress. It increased the levels of asymmetric dimethylarginine (ADMA), promoted artery calcification, and was involved in inflammation, leading to APO [9, 10]. Folate and vitamin B12 (VB12) deficiency was significantly associated with HHcy and endothelial dysfunction owing to folate and VB12 serving as co-substrates, co-factors, and vital regulators in the metabolism of Hcy [11, 12]. Supplementation of folate and VB12 could decrease serum Hcy levels in patients with HHcy [13] and also reduce oxidative stress and inflammation in pregnant rats with pregnancy-related complications [14].

Despite the harmful effects of Hcy on pregnancy outcomes, numerous related issues remain unknown. This study aimed to analyze retrospectively the relationship between subsequent events of APO and early variables of metabolic parameters, inflammatory markers, anthropometrics, and lifestyle habits in pregnant women at 11–12 weeks of gestation. It also explored the associations of subsequent events of APO with early maternal serum Hcy and folate concentrations and the VB12 status in pregnant women.

## Methods

### Study participants

This multicenter retrospective case–control study was conducted between October 2016 and March 2017 in Chengdu Women’s and Children’s Central Hospital, School of Medicine, University of Electronic Science and Technology of China, Sichuan Academy of Medical Sciences and Sichuan Provincial People’s Hospital, School of Medicine, University of Electronic Science and Technology of China, and Mianyang Hospital of Traditional Chinese Medicine. It included 563 pregnant women with APO, aged 19.33–48.25 (29.78 ± 5.97) years, and 600 non-APO pregnant women aged 21.25–46.68 (28.02 ± 4.38) years matched for age and lifestyle habits (controls).

A total of 563 APOs in this study included the following events: 234 preeclampsia without other APOs, 118 preterm birth without other APOs, 92 low birth weight without other APOs, 16 stillbirth without other APOs, 30 preeclampsia with preterm birth, 16 preeclampsia with low birth weight, 2 preeclampsia with stillbirth, 12 preeclampsia with preterm birth and low birth weight, 43 preterm birth with low birth weight.

- (1) Preeclampsia, which is defined as a new onset of hypertension and proteinuria after the 20th gestational week, or a new onset of hypertension in the absence of proteinuria but combined with hematological complications, renal insufficiency, impaired liver function, neurological symptoms, or uteroplacental dysfunction, diagnosed based on the International Society for the Study of Hypertension in Pregnancy criteria [15];
- (2) Preterm birth, which is defined as babies born alive before 37 completed weeks of gestation;
- (3) Low birth weight, which is defined by the WHO as a birth weight less than 2500 g, regardless of gestational age, caused by either preterm birth or IUGR, or a combination of both;
- (4) Stillbirth, which is defined as in utero fetal death at 20 weeks of gestation or greater, unexplained by chromosomal abnormality, anatomic malformation, or congenital infection.

While selecting 600 controls, their information of these controls was matched with that of pregnant women with APO as follows:

- (1) Similar age: The age difference between the controls and pregnant women with APO was ≤1 year, and the closest one was selected first.
- (2) Similar habits: The controls were close to pregnant women with APO in terms of smoking, drinking, and supplementation with folate and VB12.
- (3) Close delivery time: The delivery time of the controls was less than 1 week from that for pregnant women with APO, and the closest was preferred.

Women with the following causes and diseases were excluded from this study: missing data on some basic information, family history of hereditary diseases, congenital diseases, thyroid dysfunction, cancer, leukemia, or autoimmune diseases, or recent infection of TORCH, abortion, or multiple pregnancies.

### Sample collection and measurement

Fasting blood was sampled with a vacuum blood collection tube in the morning, and the blood was transferred, making it flow down the wall of the tube to minimize the mechanical disruption or turbulence that might result in hemolysis or activation. The sampled blood was vertically placed; the plasma was separated within 30 min, and the serum was separated within 60 min after blood collection. If the serum could not be separated within 60 min, the blood was vertically placed in the refrigerator at 4 °C but separated within 4 hs.

The blood was centrifuged for 10 min at 3000 rpm to separate plasma or serum. Fresh plasma was used for measuring the fasting plasma glucose (FPG) level. An aliquot of fresh serum was used for determining the levels of Hcy, folate, VB12, total cholesterol (TC), triglyceride (TG), low-density lipoprotein cholesterol (LDL-C), high-density lipoprotein cholesterol (HDL-C), uric acid (UA), and high-sensitivity C-reactive protein (hs-CRP). Another aliquot of serum was frozen and stored at − 80 °C. When the sample collection was completed, the frozen serum was brought up to room temperature by gradual thawing and centrifuged for 10 min at 3000 rpm. The serum supernatant fluid was used for determining the interleukin-6 (IL-6) level.

### Quality control

The Sichuan Homocysteine Study was conducted by the Chengdu Women’s and Children’s Central Hospital, School of Medicine, University of Electronic Science and Technology of China, the Sichuan Academy of Medical Sciences and Sichuan Provincial People’s Hospital, School of Medicine, University of Electronic Science and Technology of China, and the Mianyang Hospital of Traditional Chinese Medicine. Standard operating procedures were developed, including inclusion/exclusion criteria, project implementation, biological sample collection, storage, testing, and data collection. Moreover, the main inspection projects of all laboratories participated in the External Quality Assessment (EQA) of the National Center for Clinical Laboratories and achieved good results.

### Collection of data

The data were retrospectively collected from early pregnant women at 11–12 weeks of gestation. Complete laboratory and clinical data measured by the medical staff (doctors, technicians, nurses, and medical assistants) included serum concentrations of Hcy, folate, VB12, TC, TG, LDL-C, HDL-C, FPG, UA, IL-6, and hs-CRP; height; body weight; and blood pressure. Basic information filled in by pregnant women included ethnicity, age, gestational age, reproductive history, history of illness, family history of disease, vitamin (folate and VB12) supplementation (yes or no), and lifestyle habits such as smoking and drinking (yes or no). Non-smoking is defined as never smoked or quit smoking for more than a year, while excessive drinking is defined as drinking more than 200 mL of over 45% alcohol once a week. Information related to pregnancy, labor, birth, and maternal, fetal, and neonatal out comes was also collected. Pregnant women underwent a comprehensive examination (including some free items) when they had a health care data card in early pregnancy, benefiting from the government’s provision of free pregnancy eugenic tests for every couple of childbearing age. The data were completed, and the collection was not so difficult.

### Metabolic parameters

The concentration of Hcy was determined by the enzymatic cycling assay using a Hitachi 7600 Automatic Biochemistry Analyzer (Hitachi High-Tech Instruments Co., Ltd., Japan; reagent and calibrator, Beijing Strong Biotechnologies, Inc., China; quality control material of Liquichek Hcy, Bio-Rad Laboratories, Inc., USA). The levels of TC, TG, LDL-C, HDL-C, FPG, and UA were measured by commercial test kits using the aforementioned analyzer. The concentrations of folate and VB12 were detected by chemiluminescent immunoassay using an ADVIA Centaur XP and matched reagent, calibrator, and quality control materials (Siemens Industry, Inc., USA).

Hypercholesterolemia and hypertriglyceridemia, low HDL-C level, and high LDL-C level were defined as TC ≥ 6.22 mmol/L and TG ≥ 2.26 mmol/L, HDL-C < 1.04 mmol/L, and LDL-C ≥ 4.14 mmol/L, respectively, according to the Chinese guidelines on the prevention and treatment of dyslipidemia in adults [16]. Gestational diabetes was diagnosed when patients’ FPG was ≥5.1 mmol/L, and/or 1-h plasma glucose (1hPG) during an oral glucose tolerance test (OGTT) ≥ 10.0 mmol/L, and/or 2-h plasma glucose (2hPG) during an OGTT ≥8.5 mmol/L, according to the 2010 International Association of Diabetes and Pregnancy Study Groups recommendations on the diagnosis and classification of hyperglycemia in pregnancy [17].

The cutoff values for quartile distribution of each metabolic parameters considered in the study were Hcy (μmol/L): I < 7.03, II 7.03–7.98, III 7.99–12.60, IV > 12.60; folate (nmol/L): I < 18.31, II 18.31–21.43, III 21.44–29.25, IV > 29.25; VB12 (pmol/L): I < 255.81, II 255.81–401.28, III 401.29–546.90, IV > 546.90; TC (mmol/L): I < 4.43, II 4.43–5.29, III 5.30–5.87, IV > 5.87; TG (mmol/L): I < 1.35, II 1.35–1.57, III 1.58–1.88, IV > 1.88; LDL-C (mmol/L): I < 2.27, II 2.27–2.89, III 2.90–3.65, IV > 3.65; HDL-C (mmol/L): I < 1.22, II 1.22–1.37, III 1.38–1.62, IV > 1.62; FPG (mmol/L): I < 4.25, II 4.25–4.57, III 4.58–4.83, IV > 4.83; and UA (μmol/L): I < 271.63, II 271.63–299.09, III 299.10–372.09, IV > 372.09.

### Inflammatory markers and anthropometrics

The level of hs-CRP was measured by the noncompetitive near-infrared particle immunoassay with a matched high-sensitivity CRP Kit (IMMAGE 800 Immunochemistry System, Beckman Coulter, Inc., USA). The concentration of IL-6 was quantified by the step sandwich method with an ACCESS 2 Immunoassay System and matched a reagent and calibrator (Beckman Coulter, Inc.). Systolic blood pressure (SBP), diastolic blood pressure (DBP), body weight, and height were measured with standard techniques. The body mass index (BMI) was calculated as body weight (kg) divided by the square of height (m).

Hypertension was diagnosed when patients’ SBP was ≥140 mmHg and/or DBP ≥ 90 mmHg. Underweight, overweight, and obesity were defined as BMI < 18.5 kg/m^2^, 24.0 to < 28.0 kg/m^2^, and ≥ 28 kg/m^2^, respectively, according to the guidelines for the prevention and control of overweight and obesity in Chinese adults [18, 19]. The cutoff values for the quartile distribution of each of the inflammatory markers and anthropometrics considered in the study were hs-CRP (mg/L): I < 1.68, II 1.68–3.74, III 3.75–9.21, IV > 9.21; IL-6 (pg/mL) I < 23.64, II 23.64–37.28, III 37.29–74.57, IV > 74.57; age (year): I < 25.71, II 25.71–27.08; III 27.09–29.37, IV > 29.37; SBP (mmHg): I < 119, II 119–124, III 125–129, IV > 129; DBP (mmHg): I < 76, II 76–80, III 81–86, IV > 86; and BMI (kg/m^2^) I < 19.03, II 19.03–21.60, III 21.61–23.40, IV > 23.40.

### Statistical analysis

Data were statistically analyzed using the Statistical Package for Social Science version 19.0 (SPSS Inc., IL, USA). Continuous variables with a normal distribution were expressed as mean ± standard deviation, and the difference in data between the two groups was analyzed using the independent-samples *t* test. Continuous variables with skewed distribution were presented as median (25th to 75th percentiles), and the difference in data between the two groups was analyzed using the Mann–Whitney *U* rank-sum test. Categorical variables were presented as a percentage and analyzed using the chi-square test.

The associations of APO with each of the metabolic parameters, inflammatory markers, anthropometrics, and lifestyle habits (quantitative data expressed as quartiles and qualitative variables expressed as “yes” or “no”) were evaluated by the univariate logistic regression analysis. They were expressed as the odds ratio (OR) and its 95% confidence interval (95% CI). Variables with a *P* value < 0.25 in the univariate analysis were retained for further multivariate logistic regression analysis. The adjusted odds ratio (aOR) and its 95% CI were calculated by the multivariate logistic regression analysis (stepwise forward Wald method) to select the variables independently associated with APO. All *P* values were two-tailed, and a *P* value < 0.05 was considered statistically significant.

## Results

### Comparison of principal characteristics between pregnant women with APO and controls in early pregnancy

Compared with controls, pregnant women with APO were characterized by increased serum concentrations of Hcy, TC, TG, LDL-C, and FPG; higher value of SBP, DBP, and BMI; older age; and decreased serum levels of folate, VB12, and HDL-C (*P* < 0.05). No significant differences were found in hs-CRP, IL-6, and UA concentrations (*P* > 0.05). Moreover, pregnant women with APO had higher rates of age > 35 years, hypercholesterolemia, low HDL-C, high LDL-C, gestational diabetes, hypertension, overweight, and obesity, and lower rates of folate and VB12 supplementation compared with controls (*P* < 0.05). No significant difference was observed in the rates of smoking, excessive drinking, hypertriglyceridemia, and underweight between the two groups (*P* > 0.05). The principal characteristics of 1163 early pregnant women at 11–12 weeks of gestation according to subsequent events of APO and non-APO are reported in Table 1.

**Table 1:** Principal characteristics of 1163 early pregnant women at 11–12 gestational weeks according to subsequent events of APO and non-APO

	Whole sample (*n* = 1163)	APO (*n* = 563)	Controls (*n* = 600)	*P* value
Hcy (μmol/L)	7.98 (7.03–12.60)	8.43 (7.16–14.27)	7.79 (6.90–8.34)	< 0.001
Folate (nmol/L)	21.43 (18.31–29.25)	20.26 (17.04–27.24)	23.80 (19.48–30.24)	< 0.001
VB12 (pmol/L)	401.28 (255.81–546.90)	377.68 (233.03–541.13)	414.63 (269.85–548.17)	0.002
hs-CRP (mg/L)	3.74 (1.68–9.21)	4.15 (1.56–9.57)	3.59 (1.68–8.49)	0.149
IL-6 (pg/mL)	37.28 (23.64–74.57)	37.87 (24.29–74.57)	36.80 (22.95–73.23)	0.143
TC (mmol/L)	5.34 ± 1.20	5.46 ± 1.31	5.23 ± 1.08	0.001
TG (mmol/L)	1.92 ± 1.05	1.97 ± 1.10	1.87 ± 1.01	0.010
LDL-C (mmol/L)	3.08 ± 1.01	3.19 ± 1.09	2.97 ± 0.91	< 0.001
HDL-C (mmol/L)	1.38 ± 0.35	1.33 ± 0.39	1.43 ± 0.31	< 0.001
FPG (mmol/L)	4.79 ± 1.36	4.91 ± 1.57	4.67 ± 1.13	0.003
UA (μmol/L)	318.31 ± 58.50	318.97 ± 59.15	317.69 ± 57.92	0.708
Age (years)	28.86 ± 5.25	29.69 ± 5.94	28.02 ± 4.38	< 0.001
SBP (mmHg)	127.99 ± 16.20	129.68 ± 17.46	126.39 ± 14.77	< 0.001
DBP (mmHg)	82.08 ± 8.11	83.19 ± 8.92	81.04 ± 7.12	< 0.001
BMI (kg/m^2^)	21.82 ± 3.12	22.23 ± 3.12	21.44 ± 3.08	< 0.001
Age over 35y (%)	15.05	19.54	10.83	< 0.001
Folate supplementation (%)	88.22	82.77	93.33	< 0.001
VB12 supplementation (%)	82.55	80.11	84.83	0.034
Smoking (%)	2.06	2.49	1.67	0.326
Excessive drinking (%)	3.96	3.73	4.17	0.703
Hypercholesterolemia (%)	15.56	20.25	11.17	< 0.001
Hypertriglyceridemia (%)	20.46	22.56	18.50	0.087
Low HDL-C (%)	13.50	18.65	8.67	< 0.001
High LDL-C (%)	19.00	25.04	13.33	< 0.001
Gestational diabetes (%)	8.34	10.30	6.50	0.019
Hypertension (%)	11.26	14.21	8.50	0.002
Underweight (%)	5.16	4.80	5.50	0.587
Overweight and obesity (%)	18.40	21.31	15.67	0.013

### Relative risks of APO categories by quartiles of Hcy, folate, and VB12 in pregnant women

The relative risks of APO categories by quartiles of Hcy, folate, and VB12 in pregnant women are shown in Table 2. Compared with the lower-quartile Hcy levels, the upper-quartile Hcy levels were associated with preeclampsia (*P* < 0.001), preterm birth (*P* < 0.001), and low birth weight (*P* < 0.001). On the contrary, the lower-quartile folate concentrations were associated with preeclampsia (*P* < 0.001), preterm birth (*P* < 0.001), and low birth weight (*P* = 0.001) compared with the upper-quartile folate concentrations. However, compared with the upper-quartile VB12 levels, the lower-quartile VB12 concentrations were associated with only preterm birth (*P* = 0.008) and not with preeclampsia (*P* = 0.087) and low birth weight (*P* = 0.292). No association was observed between the upper-quartile and lower-quartile Hcy, folate, and VB12 for stillbirth.

**Table 2:** Relative risks of APO categories by quartiles of Hcy, folate and VB12 in 1163 pregnant women

APO categories	APO by quartiles of Hcy			APO by quartiles of folate			APO by quartiles of VB12		
	Odds ratio	95% CI	*P* value	Odds ratio	95% CI	*P* value	Odds ratio	95% CI	*P* value
Preeclampsia									
I quartile	1	–	–	1	–	–	1	–	–
II quartile	0.96	0.64–1.44	0.836	0.66	0.46–0.94	0.023	0.90	0.63–1.30	0.578
III quartile	1.11	0.74–1.65	0.612	0.67	0.47–0.96	0.029	0.78	0.54–1.13	0.195
IV quartile	2.55	1.76–3.69	< 0.001	0.44	0.30–0.64	< 0.001	0.72	0.50–1.05	0.087
Preterm birth									
I quartile	1	–	–	1	–	–	1	–	–
II quartile	1.34	0.81–2.20	0.255	0.91	0.63–1.43	0.949	0.71	0.47–1.07	0.100
III quartile	1.58	0.97–2.57	0.068	0.46	0.29–0.71	0.001	0.58	0.38–0.89	0.012
IV quartile	3.54	2.26–5.54	< 0.001	0.44	0.28–0.70	< 0.001	0.56	0.37–0.86	0.008
Low birth weight									
I quartile	1	–	–	1	–	–	1	–	–
II quartile	0.77	0.45–1.32	0.337	0.91	0.60–1.39	0.665	0.83	0.53–1.36	0.426
III quartile	1.03	0.63–1.69	0.912	0.56	0.35–0.90	0.016	0.81	0.51–1.31	0.362
IV quartile	2.54	1.62–3.98	< 0.001	0.43	0.26–0.71	0.001	0.78	0.49–1.24	0.292
Stillbirth									
I quartile	1	–	–	1	–	–	1	–	–
II quartile	5.07	0.59–43.67	0.140	1.20	0.36–3.99	0.761	2.02	0.50–8.16	0.323
III quartile	6.11	0.73–51.03	0.095	0.80	0.21–3.01	0.741	1.68	0.40–7.09	0.481
IV quartile	6.13	0.73–51.21	0.094	0.60	0.14–2.52	0.481	1.34	0.30–6.05	0.701

**Fig. 1:**
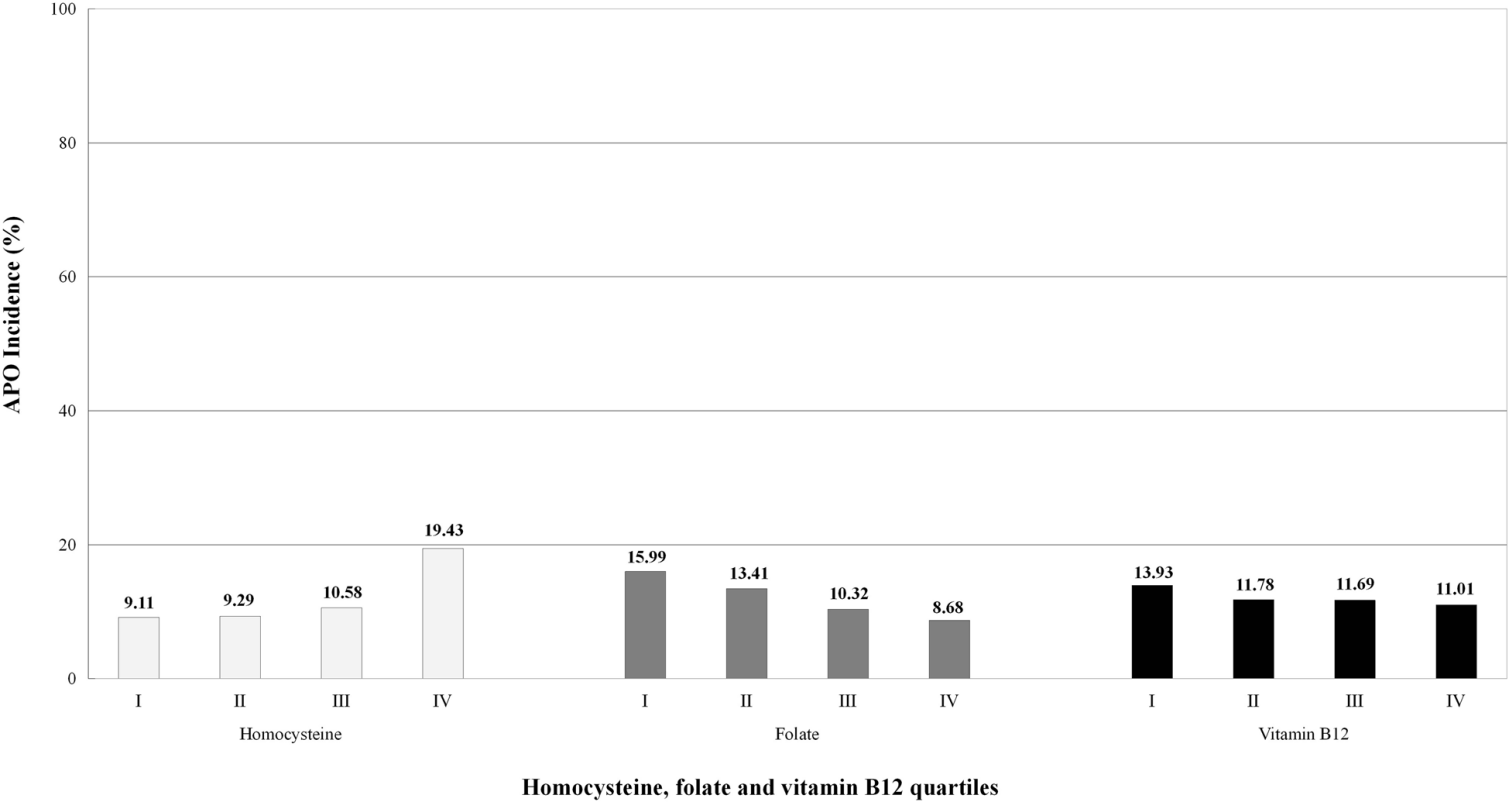
Incidence of APO by quartiles of Hcy, folate and VB12 distribution in 1163 pregnant women

**Fig. 2:**
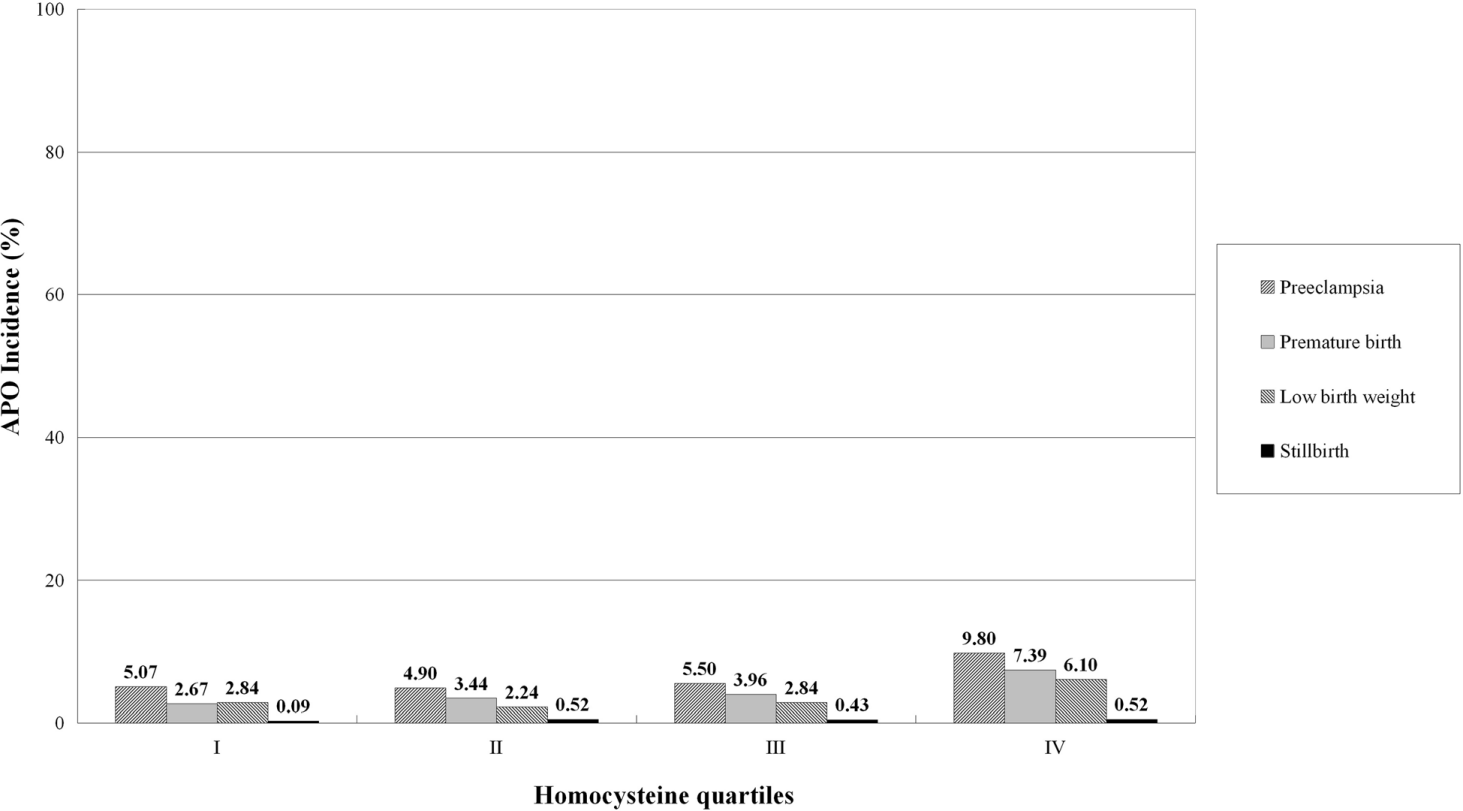
Incidence of APO categories by quartiles of Hcy distribution in 1163 pregnant women

**Fig. 3:**
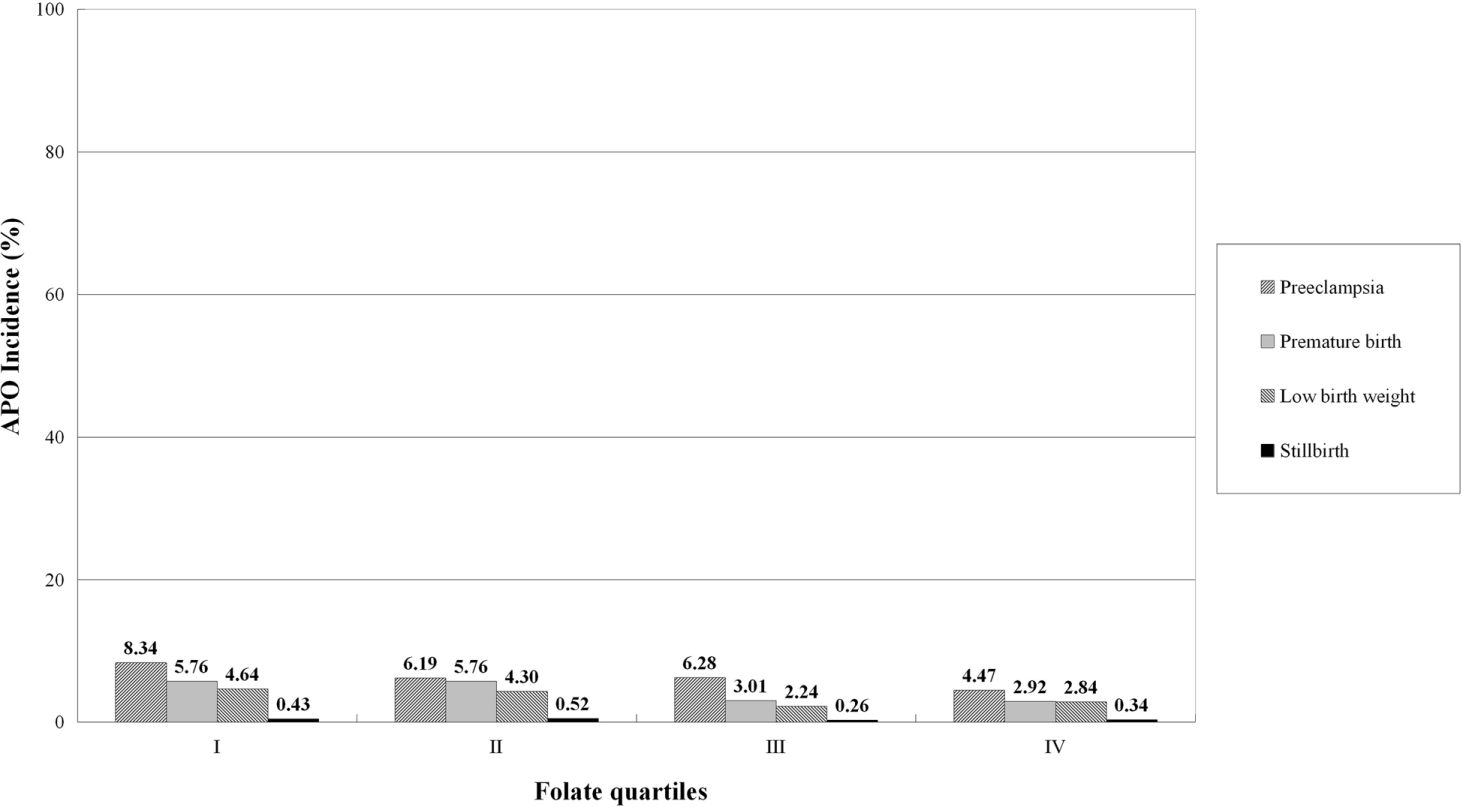
Incidence of APO categories by quartiles of folate distribution in 1163 pregnant women

**Fig. 4:**
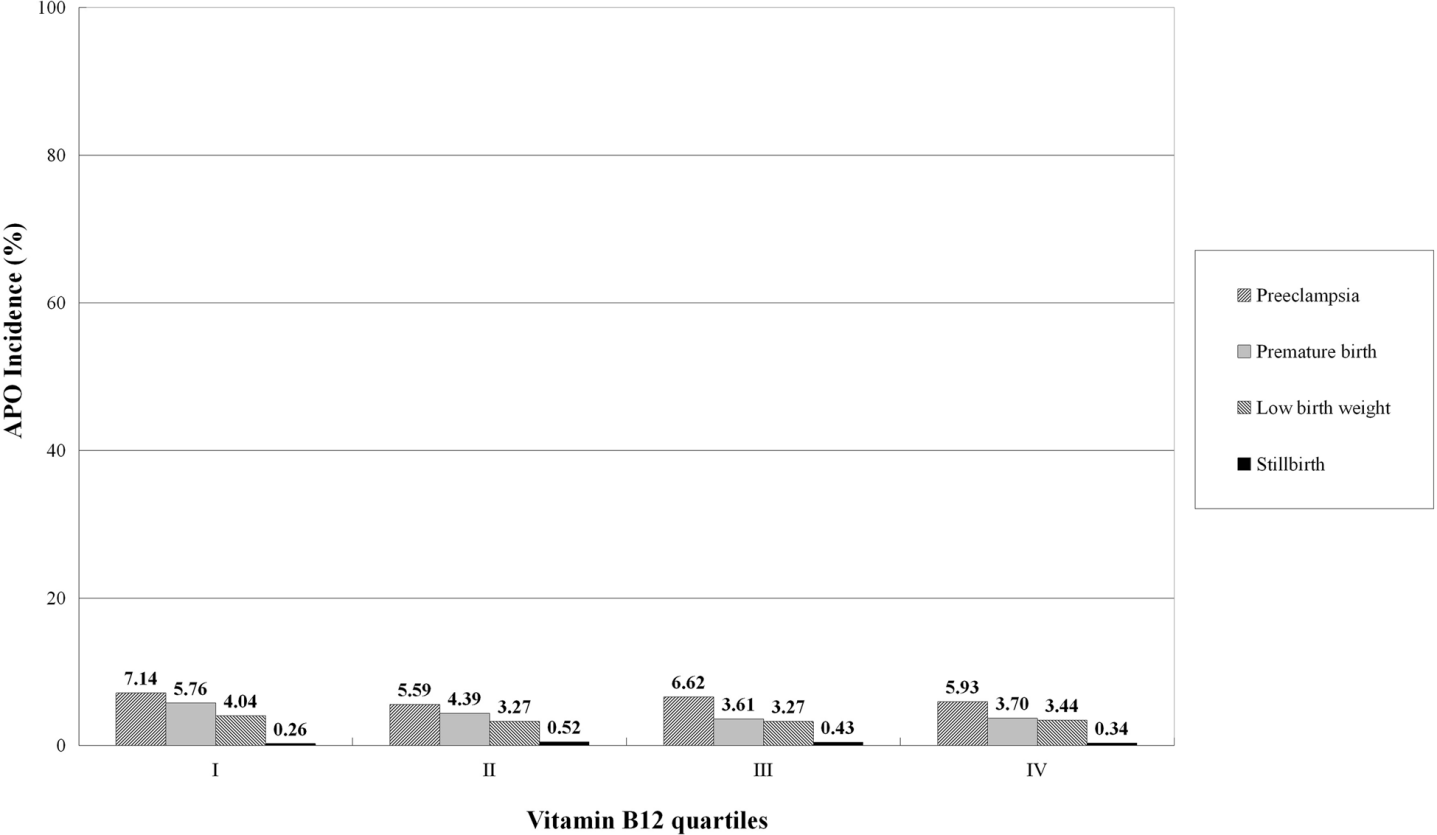
Incidence of APO categories by quartiles of VB12 distribution in 1163 pregnant women

The incidence of APO by quartiles of Hcy, folate, and VB12 distribution in 1163 pregnant women are shown in Fig. 1. The incidence of APO increased progressively from 9.11% in quartile I to 9.29% in quartile II, to 10.58% in quartile III, and to 19.43% in quartile IV of the Hcy distribution (*P* < 0.001). On the contrary, the incidence of APO decreased progressively from 15.99% in quartile I to 13.41% in quartile II, to 10.32% in quartile III, and to 8.68% in quartile IV of the folate distribution (*P* < 0.001). Similarly, the incidence of APO progressively decreased from 13.93% in quartile I to 11.78% in quartile II, to 11.69% in quartile III, and to 11.01% in quartile IV of the VB12 distribution (*P* = 0.032). Four APO categories, including preeclampsia, preterm birth, low birth weight, and stillbirth, by quartiles of Hcy, folate, and VB12 distribution in 1163 pregnant women are shown in Figs. 2, 3, and 4, respectively.

### Associations of APO with each of the early variables in pregnant women as detected by the univariate logistic regression analysis

Among qualitative variables, APO was significantly associated with folate supplementation (yes vs no, OR 0.34, 95% CI 0.23–0.51, *P* < 0.001) and VB12 supplementation (yes vs no, OR 0.72, 95% CI 0.51–0.98, *P* = 0.034). However, it was not significantly associated with smoking (yes vs no, OR 1.51, 95% CI 0.66–3.42, *P* = 0.329) and excessive drinking (yes vs no, OR 0.89, 95% CI 0.49–1.61, *P* = 0.703). Among quantitative variables, Hcy (quartile IV vs I), folate (quartile IV vs I), VB12 (quartile IV vs I), TC (quartile IV vs I), HDL-C (quartile IV vs I), SBP (quartile IV vs I), DBP (quartile IV vs I), BMI (quartile IV vs I), and age (quartile IV vs I) were significantly associated with APO (*P* < 0.05). However, hs-CRP (quartile IV vs I), IL-6 (quartile IV vs I), TG (quartile IV vs I), LDL-C (quartile IV vs I), FPG (quartile IV vs I), and UA (quartile IV vs I) were not significantly associated with APO (*P* > 0.05). The associations of APO with each of the early variables of metabolic parameters, inflammatory markers, and anthropometric variables in 1163 pregnant women, as detected by the univariate logistic regression analysis, are shown in Table 3.

**Table 3:** Univariate logistic regression analysis of the association between APO events with each of the early pregnancy variables in 1163 pregnant women

Variables	II vs I quartile				III vs I quartile				IV vs I quartile			
	Odds ratio	95% CI	Wald	*P* value	Odds ratio	95% CI	Wald	*P* value	Odds ratio	95% CI	Wald	*P* value
Hcy	1.03	0.74–1.44	0.03	0.863	1.28	0.92–1.78	2.08	0.149	6.16	4.27–8.89	94.79	< 0.001
Folate	0.65	0.47–0.91	6.36	0.012	0.40	0.28–0.55	29.48	< 0.001	0.30	0.22–0.42	47.73	< 0.001
VB12	0.71	0.51–0.98	4.29	0.038	0.70	0.50–0.97	4.63	0.031	0.63	0.45–0.87	7.69	0.006
hs-CRP	0.75	0.54–1.04	3.05	0.081	1.10	0.79–1.52	0.34	0.562	1.14	0.82–1.58	0.63	0.430
IL-6	1.23	0.89–1.70	1.55	0.213	1.32	0.95–1.83	2.75	0.097	1.19	0.86–1.65	1.08	0.300
TC	1.05	0.76–1.46	0.11	0.739	0.97	0.70–1.35	0.03	0.868	1.60	1.15–2.21	7.70	0.006
TG	1.06	0.76–1.46	0.11	0.74	1.18	0.85–1.63	0.99	0.319	1.31	0.94–1.81	2.61	0.106
LDL-C	0.77	0.55–1.01	2.51	0.114	0.97	0.70–1.35	0.028	0.868	1.33	0.96–1.84	2.90	0.089
HDL-C	0.67	0.48–0.92	5.97	0.015	0.65	0.47–0.90	6.58	0.010	0.64	0.46–0.89	7.01	0.008
FPG	1.10	0.79–1.53	0.34	0.560	1.28	0.92–1.77	2.22	0.135	1.29	0.93–1.79	2.35	0.125
UA	1.13	0.82–1.57	0.56	0.455	0.91	0.66–1.26	0.34	0.560	1.27	0.92–1.76	2.11	0.147
SBP	1.34	0.97–1.86	3.08	0.079	1.36	0.98–1.89	3.37	0.066	2.20	1.58–3.06	21.69	< 0.001
DBP	1.01	0.73–1.41	0.01	0.933	1.27	0.91–1.76	2.00	0.158	1.88	1.35–2.62	14.14	< 0.001
BMI	1.12	0.81–1.56	0.45	0.501	1.65	1.19–2.29	8.91	0.003	2.05	1.47–2.85	18.07	< 0.001
Age	0.89	0.64–1.24	0.45	0.503	1.18	0.85–1.64	0.99	0.319	1.73	1.25–2.40	10.69	0.001

### Independent associations of subsequent events of APO with each of the early variables in pregnant women as detected by multivariate logistic regression analysis

After adjusting for confounding factors, the multivariate logistic regression analysis (stepwise forward Wald method) showed that Hcy (quartile IV vs I), folate (quartile IV vs I), SBP (quartile IV vs I), DBP (quartile IV vs I), BMI (quartile IV vs I), age (quartile IV vs I), and folate supplementation (yes vs no) during early pregnancy were independently associated with subsequent events of APO (*P* < 0.05); VB12 was rejected. Of these, Hcy revealed the highest OR in all risk variables (quartile IV vs I, aOR 5.89, 95% CI 4.08–8.51), and folate showed the lowest OR in all protective variables (quartile IV vs I, aOR 0.35, 95% CI 0.25–0.50). The results of the multivariate logistic regression analysis are shown in Table 4.

**Table 4:** Multivariate logistic regression analysis (Stepwise Forward Wald) for subsequent events of APO with each of the early pregnancy variables in 1163 pregnant women

Variables	Odds ratio	95% CI	Wald	*P* value
Hcy				
I quartile	1	–	–	–
II quartile	1.03	0.74–1.45	0.04	0.852
III quartile	1.26	0.90–1.76	1.82	0.178
IV quartile	5.89	4.08–8.51	89.09	< 0.001
Folate				
I quartile	1	–	–	–
II quartile	0.71	0.50–1.00	3.96	0.047
III quartile	0.45	0.32–0.63	21.17	< 0.001
IV quartile	0.35	0.25–0.50	34.14	< 0.001
SBP				
I quartile	1	–	–	–
II quartile	1.27	0.90–1.80	1.83	0.176
III quartile	1.30	0.92–1.84	2.19	0.139
IV quartile	1.89	1.33–2.68	12.47	< 0.001
DBP				
I quartile	1	–	–	–
II quartile	1.08	0.77–1.52	0.19	0.660
III quartile	1.34	0.95–1.89	2.84	0.092
IV quartile	1.69	1.20–2.38	9.00	0.003
BMI				
I quartile	1	–	–	–
II quartile	1.14	0.81–1.60	0.53	0.467
III quartile	1.41	1.00–1.98	3.86	0.049
IV quartile	1.83	1.30–2.58	11.86	0.001
Age				
I quartile	1	–	–	–
II quartile	0.88	0.63–1.24	0.54	0.463
III quartile	1.17	0.84–1.65	0.85	0.356
IV quartile	1.52	1.08–2.14	5.75	0.016
Folate supplementation	0.55	0.35–0.86	6.72	0.010

The 15 variables included in the model were as follows: Hcy, folate, VB12, TC, TG, LDL-C, HDL-C, FPG, UA, SBP, DBP, BMI and age, folate supplementation, and VB12 supplementation according to the results of the univariate logistic regression analysis. Of these, VB12, TC, TG, LDL-C, HDL-C, FPG, UA, and VB12 supplementation were rejected by the multivariate logistic regression analysis

## Discussion

A number of factors before and during pregnancy may affect the events of APO, including maternal age [20], smoking, excessive drinking [21, 22], overweight or obesity, metabolic disorders (such as diabetes) [23, 24], and some chronic diseases such as chronic kidney disease [25], systemic lupus erythematosus [26], cardiovascular diseases [27], and so on. The results indicated that pregnant women with APO were characterized by higher Hcy, TC, TG, LDL-C, FPG, age, SBP, DBP, and BMI, and lower folate, VB12, and HDL-C concentrations, compared with controls (*P* < 0.05). In addition, pregnant women with APO had higher age (> 35 years), hypercholesterolemia, low HDL-C, high LDL-C, gestational diabetes, hypertension, overweight, and obesity compared with controls (*P* < 0.05). However, pregnant women with APO had lower folate level and VB12 supplementation compared with controls (*P* < 0.05). No significant difference was found in the rates of smoking, excessive drinking, hypertriglyceridemia, and underweight between the two groups (*P* > 0.05).

The vascular endothelium plays a pivotal role in regulating vasoconstriction and vasodilatation, coagulation and thrombosis, inflammation, and so on [28]. Endothelial dysfunction can be described as an imbalance between vasoconstrictors and vasodilators produced by the endothelium, which has significant effects on the development of placenta-mediated vascular-related diseases [7]. Hcy, as a sulfur-containing amino acid, derives from the demethylation of methionine during DNA or/and RNA methylation. It is associated with endothelial dysfunction. HHcy, as a metabolic disorder parameter, has been considered an independent risk factor for vascular-related diseases [29, 30]. Available evidence shows that HHcy may be a cause of the endothelial dysfunction provoked by oxidative stress, and elevated Hcy levels are significantly associated with preeclampsia [31], preterm birth [32], low birth weight [33], fetal death [34], IUGR [35], and so on.

In the present study, the upper-quartile Hcy concentrations were associated with preeclampsia (*P* < 0.001), preterm birth (*P* < 0.001), and low birth weight (*P* < 0.001) compared with the lower-quartile Hcy levels. The incidence of APO increased progressively from quartile I to IV of Hcy levels. After adjusting for confounding factors, the multivariate logistic regression analysis showed that, besides SBP, DBP, BMI, and age, Hcy (quartile IV vs I) was independently associated with APO. Of these, Hcy revealed the highest OR in all risk variables. The present study provided valuable evidence that higher serum Hcy concentrations during early pregnancy were independently associated with subsequent events of APO.

Maternal HHcy can increase oxidative stress, and augmented oxidative stress exerts an adverse effect on pregnancy outcome. Oxidative stress in uteroplacental tissues plays an important role in the development of placental-related diseases because of induced cellular and DNA damage and partly generated superoxide [36–39]. In women with chronic oxidative stress in the placenta, the development of the placento–decidual interface is severely impaired, leading to the early and widespread onset of maternal blood flow and major oxidative degeneration [40]. Jacobsen et al. [41] reported that more than 98% of Hcy existed in an oxidized state due to a highly reactive sulfhydryl group in Hcy, which readily self-oxidized to form a disulfide linkage with other free thiols, along with the generation of superoxide radicals as a byproduct. Maternal HHcy also increases the ADMA level, which causes the uncoupling of endothelial nitric oxide synthase (eNOS), decreased production and bioavailability of nitric oxide, and increased production of superoxide, leading to further endothelial dysfunction [42].

Folate and VB12 are two vital regulators of the metabolism of Hcy [43]. A significant association is observed between maternal lower folate concentrations and higher Hcy levels as well as an increase in the risk of preeclampsia [44]. Furness et al. [45] found that pregnant women with decreased red blood cell folate concentration and increased plasma concentrations of Hcy at 18–20 weeks of gestation had a higher risk of developing IUGR compared with controls (*P* < 0.001). They concluded that low red blood cell folate levels and high Hcy concentrations in women with the second trimester were associated with subsequent reduced fetal growth. A case–control study by Serrano et al. [46] confirmed that lower concentrations of folate were significantly associated with higher levels of Hcy in pregnant women, increasing the risk of preeclampsia.

However, folate adequacy during pregnancy was associated with a decreased risk of pregnancy-related complications, such as preterm birth, low birth weight, and fetal growth restriction [47]. A birth cohort study in a multiethnic Asian population showed that higher levels of folate in pregnant woman were associated with a lower risk of all preterm birth (OR 0.79, 95% CI 0.63–1.00) and spontaneous preterm birth (OR 0.76, 95% CI 0.56–1.04) [48]. Another birth cohort study also found that maternal higher folate concentrations were associated with a lower risk of preterm birth (aOR 0.74,95% CI 0.56–0.97) [49].

The lower-quartile folate concentrations were associated with preeclampsia, preterm birth, and low birth weight compared with the upper-quartile folate concentrations. The incidence of APO decreased progressively from quartile I to quartile IV of folate concentrations. The univariate logistic regression analysis showed that APO was significantly associated with folate (quartile IV vs I, *P* < 0.001) and VB12 levels (quartile IV vs I, *P* = 0.006), folate supplementation (yes vs no, *P* < 0.001), and VB12 supplementation (yes vs no, *P* = 0.034). After adjusting for confounding factors, the multivariate logistic regression analysis revealed that the folate level (quartile IV vs I) and folate supplementation (yes vs no) were independently associated with APO, while the VB12 level and VB12 supplementation were rejected. These findings suggested that lower serum folate concentrations during early pregnancy were independently associated with subsequent events of APO.

Increasing evidence supports that folate supplementation during pre-pregnancy and early pregnancy can reduce Hcy concentration in the bloodstream of pregnant women, thus decreasing the risk of APO. A prospective cohort study revealed that the supplementation of multivitamins containing folate in pregnant women at 12–20 weeks of gestation increased the serum folate concentration, decreased the serum Hcy level, and reduced the risk of preeclampsia (aOR 0.37; 95% CI 0.18–0.75) [50]. Another prospective cohort study also showed that the incidence of preeclampsia was lower in pregnant women with folate supplementation than in pregnant women with folate non-supplementation [51].

More recently, a large cohort study, including 240,954 pregnant women, demonstrated that folate supplementation before pregnancy was associated with 8% lower risk of preterm birth (RR 0.92, 95% CI 0.85–1.00, *P* = 0.04) and 19% lower risk of small for gestational age (SGA) birth (RR 0.81, 95% CI 0.70–0.95, *P* = 0.008) compared with controls [52]. Another large cohort study, including 200,589 singleton live birth mothers, showed that women with folate supplementation had a lower incidence of low birth weight (2.09% vs 2.27%) and SGA (5.73% vs 5.90%) compared with women without folate supplementation. It concluded that maternal daily folate supplementation significantly reduced the risks of infant low birth weight and SGA [53].

In addition, a systematic review and meta-analysis based on the UK regional population database analyzed the significance of folate supplementation in pregnancy. It revealed that the pre-pregnancy commencement of folate supplementation was associated with the reduced risk of SGA < 10th percentile (OR 0.80, 95% CI 0.71–0.90, *P* < 0.01) and SGA < 5th percentile (OR 0.78, 95% CI 0.66–0.91, *P* < 0.01). It concluded that supplementation with folate significantly reduced the risk of SGA at birth [54]. Another systematic review and meta-analysis of eight observational studies showed significantly lower odds of preeclampsia in pregnant women with folate supplementation compared with those without folate supplementation (OR 0.78, 95% CI 0.63–0.98) [55].

In a multicenter study by Wen et al. [56], which was a randomized and double–blind study, including 2301 pregnant women with at least one high risk factor for preeclampsia (1144 to the folate group and 1157 to the placebo group), preeclampsia occurred in 169/1144 (14.8%) women in the folate group and 156/1157 (13.5%) in the placebo group. No differences were found between the two groups (RR = 1.10, 95% CI 0.90–1.34, *P* = 0.37). This study also showed that supplementation with 4.0 mg/day folate beyond the first trimester did not prevent preeclampsia in women at high risk of this condition. It indicated that more evidence is needed to explore the relationship between folate and APO.

The present study had several limitations. First, it did not evaluate the efficacy and safety of the use of high doses of folate supplementation during early pregnancy for APO prevention because it was a retrospective case–control study. In addition, it only measured the concentration of VB12, but not its activity. It is necessary to further determine the activity of VB12 so as to provide a more reliable assessment of VB12 status in early pregnancy. Second, the quantitative parameters were expressed as the quartiles during data analysis in this study, leading to significant bias (versus tertiles, quintiles, etc.). Third, the BMI threshold for overweight and obesity in this study was defined as 24.0–27.9 kg/m^2^ and ≥ 28 kg/m^2^, respectively, because the participants were all Chinese, which differed from the usual cutoff of 25–29.9 kg/m^2^ and 30 kg/m^2^, respectively, in the general Western population. The prevalence of overweight and obesity, as shown in Table 1**,** might have slightly changed if the cutoff value appropriate for the Western population was used. However, the result that APO was independently associated with BMI (quartile IV vs I, aOR 1.83, 95% CI 1.30–2.58, *P* = 0.001) did not change because BMI quartiles without threshold were used in the logistic regression analysis. Furthermore, APO and control groups had a very low percentage of smoking (2.49% vs 1.67%) and excessive drinking (3.73% vs 4.17%), leading to distorted results of the logistic regression analysis due to sampling bias.

## Conclusions

This study demonstrated that higher Hcy concentration as a risk factor and lower folate level as a protective factor during early pregnancy were associated with subsequent events of preeclampsia, preterm birth, and low birth weight. The incidence of APO increased progressively from quartile I to IV of Hcy levels, but decreased progressively from quartile I to IV of folate concentrations. Hcy (quartile IV vs I) and folate levels (quartile IV vs I) and folate supplementation (yes vs no) during early pregnancy were independently associated with subsequent events of APO. However, no association was found between VB12 and APO. Supplementation with folate in early pregnancy may reduce APO.

## Data Availability

The data sets analyzed during the current study are available from the corresponding author on reasonable request.

## Abbreviations

1hPG: 1-h plasma glucose
2hPG: 2-h plasma glucose
95% CI: 95% confidence interval
ADMA: Asymmetric dimethylarginine
aOR: adjusted odds ratio
APO: Adverse pregnancy outcome
BMI: Body mass index
DBP: Diastolic blood pressure
eNOS: endothelial nitric oxide synthase
EQA: External Quality Assessment
FPG: Fasting plasma glucose
Hcy: Homocysteine
HDL-C: High-density lipoprotein cholesterol
HHcy: Hyperhomocysteinemia
hs-CRP: high-sensitivity C-reactive protein
IADPSG: International Association of Diabetes and Pregnancy Study Groups
IL-6: Interleukin-6
ISSHP: International Society for the Study of Hypertension in Pregnancy
IUGR: Intrauterine growth restriction
LDL-C: Low-density lipoprotein cholesterol
OGTT: Oral glucose tolerance test
OR: Odds ratio
SBP: Systolic blood pressure
SD: Standard deviation
SGA: Small for gestational age
TC: Total cholesterol
TG: Triglyceride
UA: Uric acid
VB12: Vitamin B12
WHO: World Health Organization
